# Sustainably Cultivating and Harvesting Microalgae through Sedimentation and Forward Osmosis Using Wastes

**DOI:** 10.1021/acsomega.1c01474

**Published:** 2021-06-25

**Authors:** Hannah R. Molitor, Alyssa K. Schaeffer, Jerald L. Schnoor

**Affiliations:** Department of Civil and Environmental Engineering, University of Iowa, 103 S. Capitol Street, Iowa City, Iowa 52242, United States

## Abstract

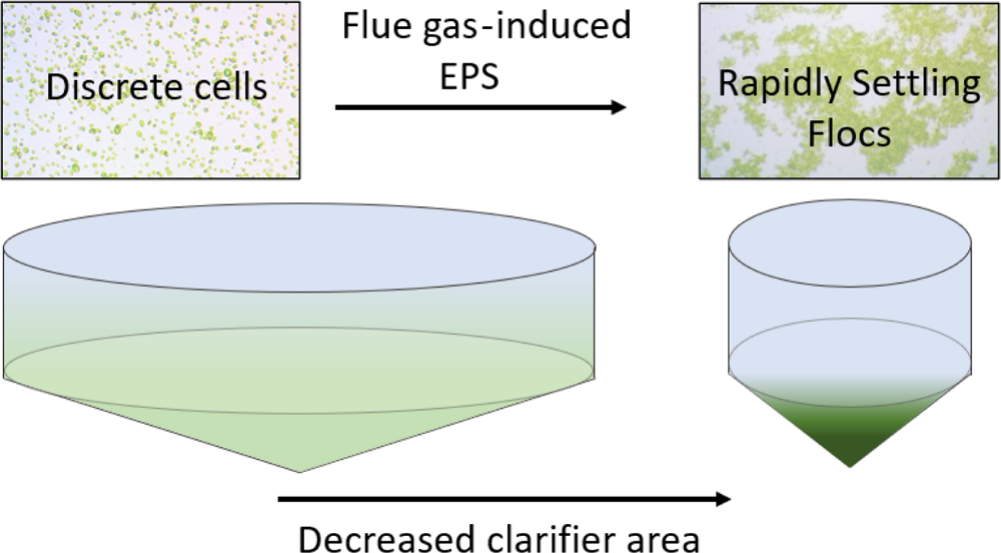

Cost-effective nutrient sources and dewatering are major obstacles to sustainable, scaled-up cultivation of microalgae. Employing waste resources as sources of nutrients offsets costs for nutrient supplies while adding value through simultaneous waste treatment. Forward osmosis (FO), using simulated reverse osmosis brine, is a low-energy membrane technology that can be employed to efficiently harvest microalgae from a dilute solution. In this study, *Scenedesmus obliquus*, a green microalga, was cultivated with a fertilizer plant wastewater formula and simulated coal-fired power plant flue gas and then separated through either FO, with reverse osmosis reject model water as the draw solution, or sedimentation. Microalgal batches grown with simulated wastewater removed NH_4_^+^ within 2 days and reached nitrogen and phosphorus limitation simultaneously on Day 5. Sparging with the flue gas caused *S. obliquus* to produce significantly greater quantities of extracellular polymeric substances (30.7 ± 1.8 μg mL^–1^), which caused flocculation and enhanced settling to an advantageous extent. Five-hour FO trials showed no statistically significant difference (*p* = 0.65) between water fluxes for cultures grown with simulated flue gas and CO_2_-supplemented air (3.0 ± 0.1 and 3.0 ± 0.3 LMH, respectively). Reverse salt fluxes were low for all conditions and, remarkably, the rate of reverse salt flux was −1.9 ± 0.6 gMH when the FO feed was culture grown with simulated flue gas. In this work, *S. obliquus* was cultivated and harvested with potential waste resources.

## Introduction

Sustainable and economical microalgal cultivation is an opportunity to treat wastes, sequester CO_2_, and produce biomass commodities while managing finite resources responsibly. Although microalgae are metabolically efficient, adaptable, and rapidly produce biomass year-round, further technological advancement is necessary to implement full-scale, cost-effective operations.^1,2^ Successful microalgal biomass production can be threatened by high capital costs, inefficient temperature regulation, evaporation losses, high land footprints, contamination, and high-energy dewatering and harvesting processes. Using waste resources for cultivation and low-energy technologies for harvest would improve the economic feasibility of microalgal biomass production, especially as we come to recognize a greater financial value in greenhouse gas mitigation and recovery of finite nutrient resources.

### Separation of Microalgae from Dilute Culture Solutions

Harvesting microalgae from dilute solutions (0.02–0.5% w/v)^1^ in a cost- and energy-efficient manner is a major hurdle to the industry. Common particle-separation techniques include sedimentation, flocculation, flotation, filtration, centrifugation, or a combination of methods, often in two stages.^3^ The energy requirement for sedimentation is low, but microalgae have low settling rates; a *Chlorella* sp. was reported to have a settling rate of 0.1 m d^–1^.^4^ Higher settling rates are uncommon without chemical or biological additives or contaminants that cause flocculation. Filtration, flotation, and centrifugation can achieve rapid separation with high biomass recovery rates, but each have high energy requirements. High-energy or chemically facilitated separation techniques may also negatively affect the microalgal cell structure.^5,6^ Enhanced sedimentation through autoflocculation or low-energy filtration through forward osmosis (FO) may offer more economical and sustainable methods for microalgal harvest.^7^

### Enhanced Settling

Flocculation is widely accepted as a method to enhance settling rates for microalgal harvest, whether chemically or biologically caused.^8^ However, chemical flocculants are not generally suitable for media or sludge recycling.^3^ Bacteria, yeast, or fungi can cause flocculation or form pellets that settle rapidly but change the composition of the final biomass product. Under some stress conditions (e.g., high or low pH, toxins, salinity), there are species of microalgae (including *Scenedesmus* species) that produce extracellular polymeric substances (EPSs) for protection, autoflocculate, and consequently settle more rapidly.^9,10^ Though stress can compromise biomass productivity, increased EPS concentrations in the media can be beneficial for enhanced settling.

Settling can be described by four distinct sets of characteristics, type I through IV.^11^ Type I settling occurs when discrete particles settle individually from dilute solutions with no aggregation; a linear settling rate can typically be determined. Type II settling occurs when particles in dilute solutions flocculate as they settle and change size and shape, which causes the settling rate to change (often increasing) with depth. When settling is hindered, it is known as type III settling, in which particles form low-density aggregates which have settling rates that decrease with depth. Type IV settling occurs when the mass of settled particles compresses the bulk below them.

### FO for Sustainable Microalgal Harvesting

FO depends on an osmotic gradient for water flux from the feed solution to the draw solution,^12^ rather than the high hydraulic pressures necessary for other membrane separations. When applied to dilute microalgal cultures, FO can dilute a waste brine, concentrate the microalgal culture, avoid fouling, and reduce the volume of culture requiring high-energy dewatering.

Membrane fouling that does occur during FO is reversible and can be removed via physical cleaning (i.e., hydraulic flushing).^13^ However, microalgal aggregation, reduced water flux, and biomass loss have been caused by reverse salt flux of Mg^2+^ and Ca^2+^ from the draw solution in previous FO studies for microalgal separation.^14^ While enhanced settling is advantageous to separation via sedimentation, those same characteristics can lead to the collection of biomass on the membrane during FO separation.^15^

Reverse osmosis (RO), a membrane separation process dependent on high hydraulic pressure, is used to remove contaminants and pathogens from drinking water, purify water for biofuel production, treat industrial effluents, concentrate food liquids, and desalinate seawater.^16,17^ RO yields purified water and the balance is RO reject water, a high ionic strength waste suitable for FO draw solution. Because the discharge of high-ionic-strength wastes is environmentally damaging, dilution through FO offers a possibly advantageous low-energy solution for brine treatment by decreasing the ionic strength of RO reject water.^18^ In full-scale operations, repurposing a high-salinity waste stream as the FO draw solution removes the cost of solute purchase and separation for recycle.

Reverse salt flux can be problematic because the salts from the draw solution could contaminate the microalgal culture and further reduce the osmotic pressure gradient, even as the water flux is also decreasing the gradient. Generally, FO studies use inorganic salts as the draw solution because they are inexpensive and have potential for high osmotic pressure. However, the small ionic radius and low charge of mono- and divalent ions risk a high-reverse-salt flux, on the order of approximately 5 to 20 g m^–2^ h^–1^ when paired with the deionized (DI) water feed.^19,20^ In this scenario, where the draw solution is RO-concentrated seawater, reverse salt flux would not contribute any harmful ionic species to the microalgal culture but could slow the process of concentrating the biomass. Nonetheless, the advantages of treating two streams simultaneously with low energy demand could outweigh these concerns.

### Waste as a Source of Nutrients for Microalgae

Of the nutrients required by microalgae, the quantity and source of nitrogen is most impactful for growth and cell composition.^21^ Cell protein, carbohydrate, and lipid fractions are dependent on available nutrient concentrations, but the metabolic response is species-dependent and most strongly influenced by nitrogen.^22^ Nitrate is the most common nitrogen source for synthetic media.

Ammonium is a favorable nitrogen source because it requires less energy for uptake^23^ but can be detrimental to cultivation because it can decrease the growth rate of microalgae.^21^ The p*K*_a_ of ammonia/ammonium is 9.25 at 25 °C, and the cultivation medium pH should be regulated to avoid ammonia toxicity at high pH values.^24^ As in conventional agriculture, ammonia would likely be the nitrogen supply used for the production of nitrogen fertilizers for full-scale microalgal cultivation.^25^ A more sustainable approach would be to cultivate microalgae on the wastewater of nitrogen fertilizer plants. Producing valuable microalgal biomass from wastewater could offset the cost of conventional wastewater treatment, but biomass productivity could be inhibited by wastewater toxicants.^26,27^

Both nitrate and ammonium are present in fertilizer plant wastewater, and microalgae use both to grow.^9^ However, the simultaneous presence of ammonium and nitrate creates conditions for counter-repression.^28^ If ammonium is present, nitrate uptake will be repressed and if high concentrations of nitrate are present, ammonium uptake is repressed.^29−31^ When ammonium and nitrate are both present in concentrations that can sustain microalgal growth, microalgae will preferentially use ammonium.^28^ To use oxidized nitrogen species in cell processes, microalgae must first reduce nitrate or nitrite to ammonium through either nitrate reductase and then nitrite reductase or nitrite reductase, respectively.^32^ In certain species, NO_2_^–^ will also be preferentially used before NO_3_^–^, and these nitrogen species will also create an environment for counter-repression.

Limited quantities of nitric oxides are available to microalgae from flue gases but slow diffusion rates, slow oxidation rates, and/or low concentrations decrease their utility.^33^ In most cases, the quantity of nitrogen supplemented by flue gases would be insignificant relative to the quantity of nitrogen required to sustain microalgal growth. On the other hand, sufficient sulfate for microalgal growth will be rapidly accumulated from the oxidation of SO_2_ in flue gas.^34^

Though it is approximately 1% or less of the dried microalgal biomass, phosphorus is also an essential nutrient. Microalga N/P ratios are wide-ranging, including within a single species, depending on environmental conditions and growth stage.^35^ From biomass elemental composition analyses, the N/P molar ratio of *Scenedesmus obliquus* grown to stationary phase with a N- and P-rich medium and simulated power plant flue gas was 14:1.^34^

Here, we show the potential for microalgal cultivation and harvest using waste resources. When *S. obliquus* was cultivated with simulated coal-fired power plant flue gas and simulated fertilizer plant wastewater, simultaneous N- and P-limitation was achieved in 5-day batches. FO, with simulated RO reject water as the draw solution, and sedimentation were studied as sustainable, low-energy separation techniques. EPS production was greatly stimulated by the combination of simulated flue gas and simulated wastewater, so much so that the microalgae formed good flocs with rapid settling rates but that also settled within the lower-flow area inside the FO cell. Water flux rates of 3 L m^–2^ h^–1^ were achieved and reverse salt flux was low for control and CO_2_-supplemented air cultures. Remarkably, the salt flux was not “reverse” for the culture grown with simulated emissions, and instead low concentrations of ions moved from the feed solution to the draw solution (negative reverse salt flux occurred).

## Results and Discussion

### Microalgal Growth and Nutrient Utilization

**Figure 1:**
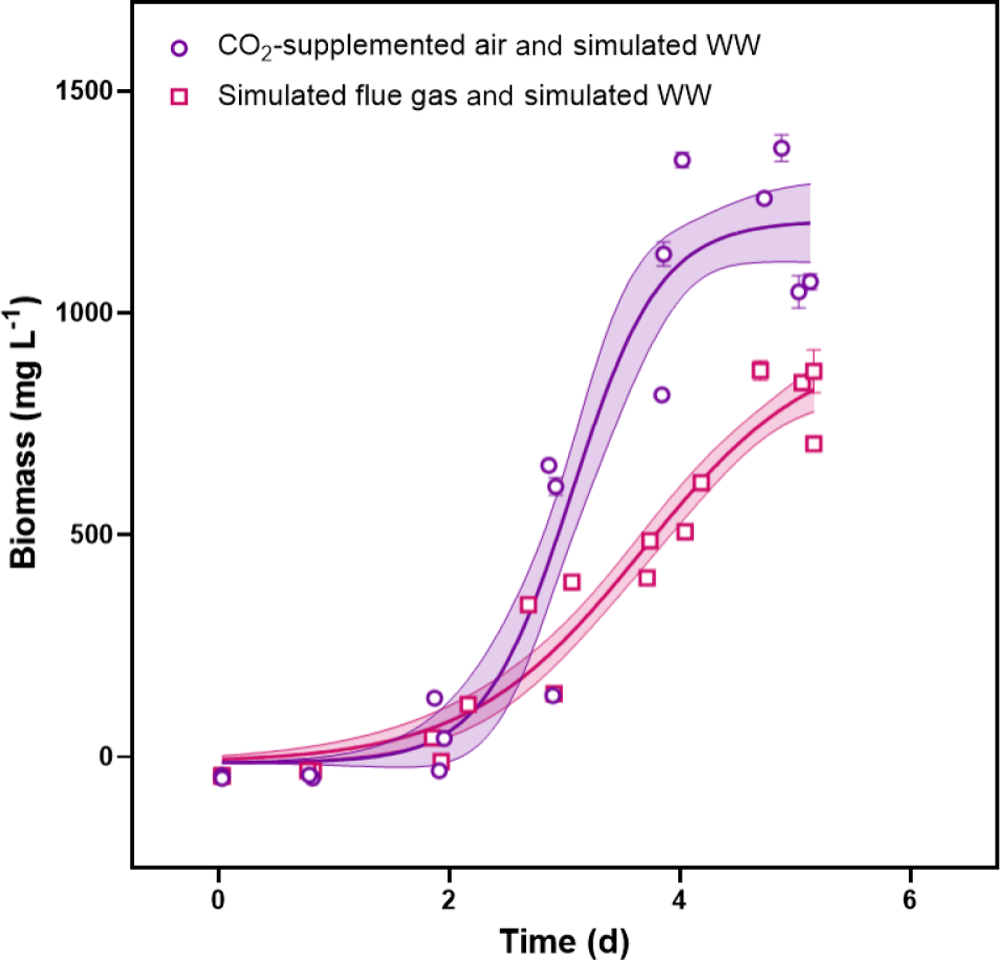
Biomass productivity of *S. obliquus* growth with (1) simulated coal-fired power plant emissions and simulated fertilizer plant wastewater and (2) CO_2_-supplemented air and simulated fertilizer plant wastewater. All points for both triplicate experiments are shown. Vertical error bars represent ±1 standard deviation, and the shaded regions represent 95% confidence intervals on the modeled curves.

To investigate the effect of nutrients from waste sources (both power plant emissions and fertilizer plant wastewater) on microalgal biomass productivity, *S. obliquus* was grown under the following three conditions: (1) simulated coal-fired power plant emissions (12% CO_2_, 6% O_2_, 500 ppm SO_2_, 500 ppm CO, and 200 ppm NO_2_) and simulated fertilizer plant wastewater, (2) 12% CO_2_-supplemented air and fertilizer plant wastewater, and (3) 12% CO_2_-supplemented air and 3N-BBM (BBM: Bold’s basal medium; control, see Supporting Information, Figure S1). Microalgae reached stationary phase at Day 5 (Figure 1). Two of the three flue gas trials reached stationary phase on Day 5, and one trial ended early on Day 4; biomass concentrations would not have increased past Day 5 because N and P were depleted. The average overall biomass productivity for the flue gas triplicate trials was 160 ± 20 mg L^–1^ days^–1^, whereas only wastewater triplicates produced 276 ± 16 mg L^–1^ d^–1^. The maximum biomass productivities of coal-fired power plant emissions and fertilizer plant wastewater experiments and of CO_2_-supplemented air and fertilizer plant wastewater experiments were 350 ± 40 and 850 ± 30 mg L^–1^ d^–1^, respectively.

In a previous study, using 3N-BBM (125 mg L^–1^ N), flue gas promoted greater biomass productivity rates than CO_2_-supplemented air.^34^ In this study, using two nitrogen species at a lesser initial concentration (47 mg L^–1^ N), flue gas caused lower biomass productivity rates. We hypothesize that the stress of exposure to acidic and toxic flue gas components (CO, NO_2_, and SO_2_) was compounded with the stress of ammonia toxicity in microenvironments. 1 N NaOH base was added at the beginning of each batch trial and intermittently throughout for pH control, which likely created microenvironments that exposed cells to locally high pH and NH_3_, the reaction product of OH^–^ and NH_4_^+^. Counter-repression between nitrate and ammonium uptake enzymes may have contributed to reduced growth rates also.

The hypothesis of attributing lower growth rates to stress was supported by the significantly increased EPS concentrations in cultures grown with both flue gas and wastewater (see the section In EPS and Modeled Bulk Settling) and the decrease of nitrate removal rates in cultures grown with wastewater relative to the control.

**Figure 2:**
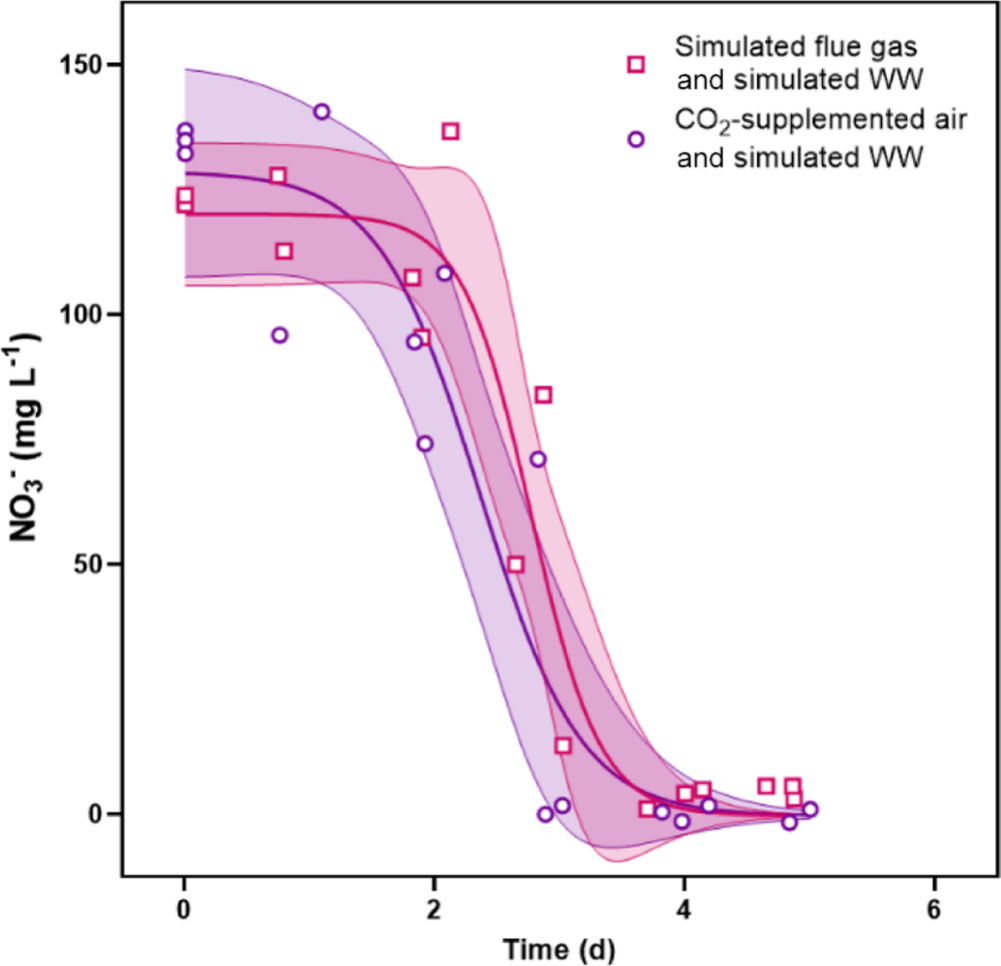
Removal of NO_3_^–^ from culture medium over time for experiments with simulated wastewater and either CO_2_-supplemented air or simulated flue gas. Shaded regions represent 95% confidence intervals on the modeled curves.

**Figure 3:**
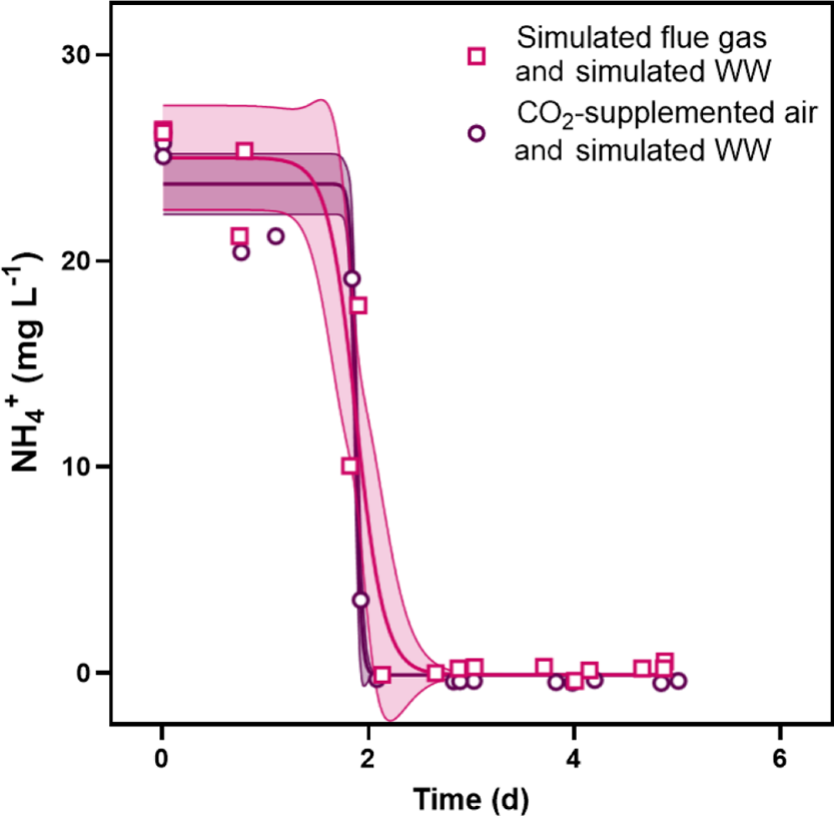
Removal of NH_4_^+^ from culture medium over time for experiments with simulated wastewater and either CO_2_-supplemented air or simulated flue gas. Control cultures did not contain NH_4_^+^. Shaded regions represent 95% confidence intervals on the modeled N-depletion curves.

Nitrate and ammonium depletion curves showed that NH_4_^+^ was preferentially removed before NO_3_^–^; NH_4_^+^ was removed by Day 3 and NO_3_^–^ was removed by Day 5 (Figures 2 and 3). Ammonium was removed much more rapidly from trials with only wastewater than trials with flue gas and wastewater. Maximum removal rates for NH_4_^+^ were 225 ± 12 and 46 ± 5 mg L^–1^ d^–1^, respectively. Flue gas had the opposite, and much less dramatic, impact on NO_3_^–^ removal rates. Maximum removal rates for NO_3_^–^ were 80 ± 10 and 110 ± 10 mg L^–1^ d^–1^, respectively, in trials with only wastewater than in trials with flue gas and wastewater. Nitrate was completely removed by Day 5 under both conditions. The maximum nitrogen removal rates for both dual-nitrogen source conditions were significantly less than the control nitrate removal rate (220 ± 20 mg L^–1^ d^–1^ NO_3_^–^) (see Supporting Information, Figure S2).

**Figure 4:**
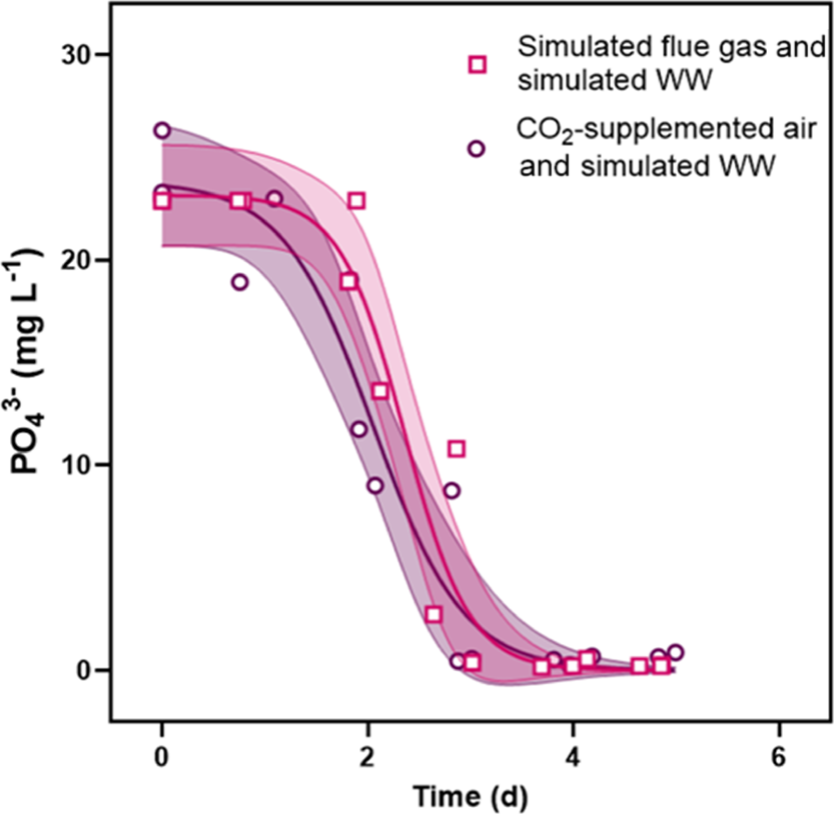
Removal of PO_4_^3–^ from culture medium over time for experiments with simulated wastewater and either CO_2_-supplemented air or simulated flue gas. Shaded regions represent 95% confidence intervals on the modeled P-depletion curves.

Phosphate was removed more rapidly from trials with simulated flue gas and wastewater than trials with only simulated wastewater (Figure 4). Maximum removal rates for PO_4_^3–^ were 18.4 ± 1.1 and 13.6 ± 0.9 mg L^–1^ d^–1^, respectively. For both conditions, PO_4_^3–^ was removed by Day 5, the same time at which nitrogen was completely depleted. In control cultures, which had N/P ratios much lower than 14:1, the system was nitrogen limited, and a large quantity of PO_4_^3–^ remained even as the culture reached stationary phase. The maximum phosphate removal rate from the control cultures was 33 ± 2 mg L^–1^ d^–1^ PO_4_^3–^ (see Supporting Information, Figure S3).

**Figure 5:**
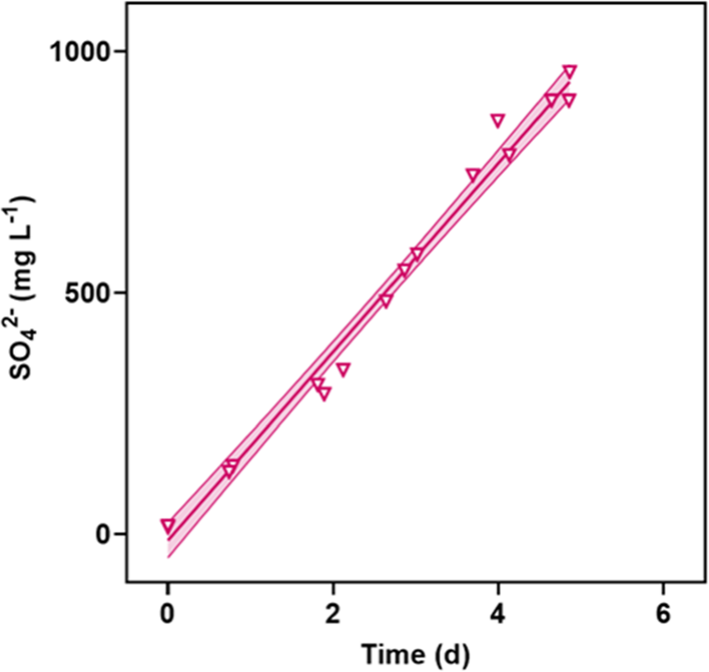
Accumulation of SO_4_^2–^, from the rapid oxidation of the sparged SO_2_ gas, in the culture medium during experiments with simulated flue gas and simulated fertilizer plant wastewater. *y* = 195.5*x* – 12.6. *R*^2^ = 0.9875. Shaded area represents the 95% confidence interval on the linear regression.

Sulfate accumulated linearly in the culture medium when it was sparged with simulated flue gas containing SO_2_. In this study, SO_2_ was rapidly oxidized to SO_4_^2–^ in quantities that exceeded the nutrient requirements of microalgae (Figure 5). The photobioreactor was an oxidizing environment: the flue gas was 6% O_2_; Mn^2+^, Co^2+^, and Fe^2+^ were available to act as catalysts; and the presence of oxidants, including hydroxyl radical, hydrogen peroxide, NO_2_, and ozone was likely.

At full scale, the accumulation of SO_4_^2–^ in the treated wastewater would be determined by the SO_2_ concentration in the flue gas, the sparging rate of the flue gas, and the hydraulic retention time of the media. To avoid enormous land footprints for microalgal treatment systems while maintaining treatment capacity, the solids retention time and hydraulic retention time should be differentiated by recycling some of the microalgae solids.^36^ Hydraulic retention times would typically be on the order of a few hours.^37^ Under these conditions, 0.07 vvm sparging and 500 ppm SO_2_, any hydraulic retention time less than one day would preclude the accumulation of sulfate to concentrations that would be problematic for treated wastewater discharge (24-h prediction: 180 mg L^–1^, less than the secondary drinking water standard: 250 mg L^–1^).^38^

### In EPS and Modeled Bulk Settling

**Figure 6:**
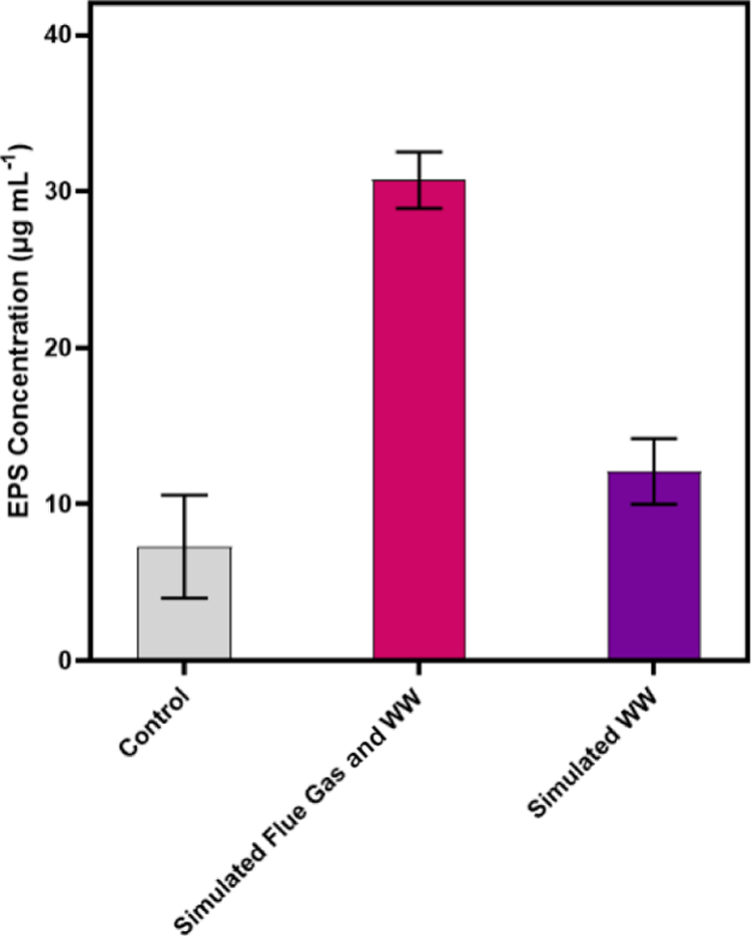
EPS concentration comparison among *S. obliquus* cultures grown with CO_2_-supplemented air and 3N-BBM (control), simulated fertilizer wastewater and simulated flue gas, and CO_2_-supplemented air and simulated fertilizer wastewater. Error bars represent ±1 standard deviation.

EPS production as a cause of flocculation was confirmed through the anthrone–sulfuric acid method for polysaccharides. At the time of harvest, Day 5, *S. obliquus* cultivated with simulated flue gas and wastewater had produced 30.7 ± 1.8 μg mL^–1^ EPS and *S. obliquus* cultivated with CO_2_-supplemented air and simulated wastewater had produced 12.1 ± 2.1 μg mL^–1^ EPS (Figure 6). The control culture accumulated 7 ± 3 μg mL^–1^ EPS. Bacterial contamination, which could cause flocculation, was neither evident in phase contrast microscopy at 100× magnification nor in agar streak plates.

**Figure 7:**
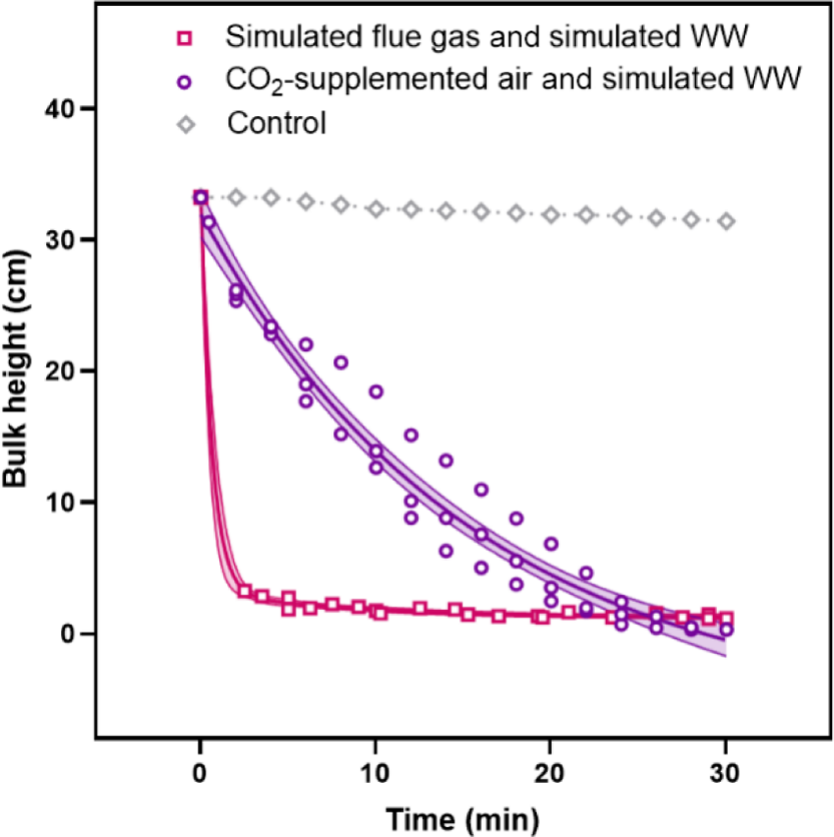
Bulk settling process, through coagulation and compaction, of microalgae grown on simulated power plant emissions or with simulated fertilizer plant wastewater. The model error was represented with a 95% confidence interval. Settling of control microalgae was not observed within the 30 min period.

Bulk settling caused by flocculation induced by ammonia or flue gas stress was modeled with one-phase and two-phase exponential decays, respectively (eqs 1 and 2). For *S. obliquus* cultivated with flue gas, the region of coagulation and rapid settling occurred across the upper 30.2 ± 0.5 cm, the region of compaction was at 1.8 ± 0.4 cm and the minimum compacted bulk height was predicted to be 1.2 ± 0.2 cm (Figure 7). The settling rate for the flue gas condition was approximated to be 11.6 cm min^–1^ (average linear rate across the first 30.2 cm region of rapid settling). According to eq 1, *S. obliquus* cultivated with simulated wastewater would require 21.9 min to settle the first 30.2 cm.
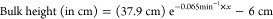
1
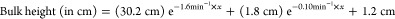
2

The control settled slowly as discrete particles at a rate of 0.62 ± 0.03 mm min^–1^ during the 30 min trials. The average fraction of biomass that settled in bulk after cultivation with simulated flue gas or with simulated wastewater was 60% ± 20% and 43% ± 5%, respectively. Previous studies, where settling depended on self-flocculating microalgal species, achieved 25% to 40% biomass recovery during a harvest period of 3 h.^3,39^ Interestingly, a previous study deemed that *S. obliquus* did not flocculate sufficiently for use as a bioflocculant.^39^ However, in this study, the simulated flue gas with wastewater (pink squares in Figure 7) clearly had the highest EPS concentrations and settled the fastest.

The thickened, emissions-sparged culture achieved 60% biomass recovery and 4.5% ± 1.5% solids in the settled bulk. In studies of bioflocculation or chemical flocculation and sedimentation, 34% to 99% and 67% to 99% biomass recovery was achieved, respectively, and 0.5% to 3% solids was typical.^3,39−48^ However, autoflocculation avoids chemical or biological inputs, which can contaminate the product, contaminate media which could otherwise be recycled, necessitate production of synthetic materials, and increase production cost.^49^ An additional advantage of the results of this study was that flocculation was facilitated during cultivation, which avoids an added harvest period for the flocculation action of a biological or chemical flocculating agent.

In wastewater treatment, secondary clarifiers are used to concentrate then remove or recycle sludge. Settling characteristics are critical for clarifier design and are determined by the solids concentration and the settling type (I through IV). In microalgal cultivation, the same sedimentation strategy can be used for microalgal harvest or recycle, if settling rates are sufficiently rapid.

From the settling experiments, biomass grown with flue gas initially settled as type II then type IV, skipping the type III hindered settling that is common in secondary clarifiers. Within the region from 33.3 cm to 3.1 cm, *S. obliquus* rapidly flocculated and settled in relatively dense flocs. The flocs had sufficient density to cause compaction of the bulk biomass accumulated at the bottom of the graduated cylinder for the remaining 27.5 min of the 30-min settling trials. The bulk biomass was compacted from a height of 3.1 cm to 1.8 cm during this period. Biomass grown with CO_2_-supplemented air settled according to type III characteristics. The flocs were visibly less dense and would form loose attachments with other flocs as they settled. The settling rate decreased with depth. Control biomass settled slowly at a linear rate as discrete particles in a dilute solution (type I). Clarifier sizing depends on a design surface overflow rate (SOR), which represents the volumetric flow rate into the clarifier divided by the clarifier surface area but is determined by the solids settling rate. For equal flow rates, the clarifier surface area required to separate microalgae cultivated with flue gas from solution would be approximately 200 times less than that required for the control biomass. The enhanced settleability of *S. obliquus* grown with flue gas moves sedimentation as a microalgal harvesting strategy from infeasible to feasible.

### FO Dewatering Efficiency

**Figure 8:**
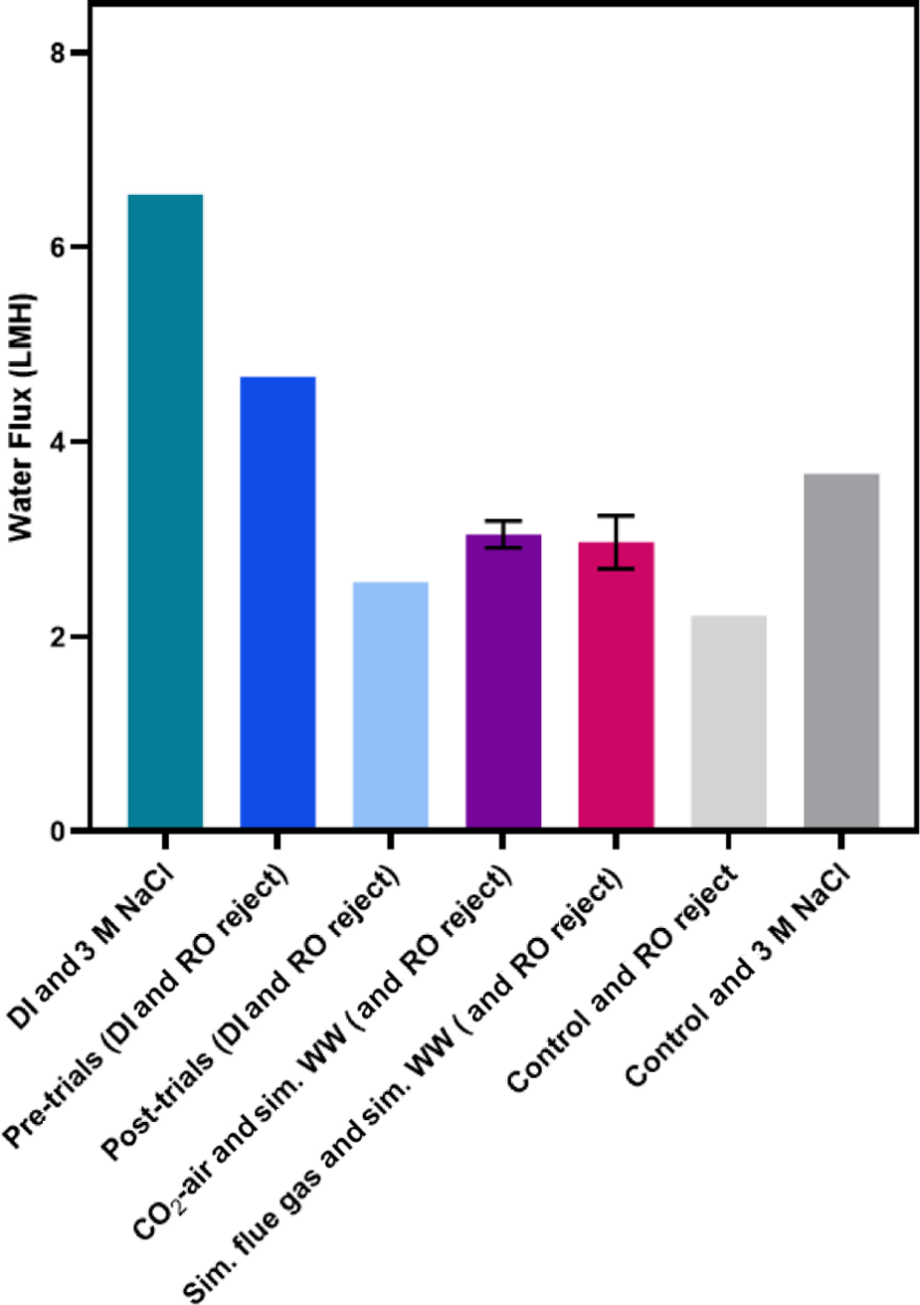
Water flux across the FO membrane from the feed to draw for combinations of DI water or*S. obliquus* cultures as the feed, and 3 M NaCl or simulated RO reject water as the draw. Error bars represent ±1 standard deviation.

The FO process concentrated the dilute microalgal culture, which reduced the volume requiring further separations by 30% in 5 h. Water recovery was examined for DI water, control cultures grown with CO_2_-supplemented air and 3N-BBM, cultures grown with simulated wastewater and CO_2_-supplemented air, and those grown with simulated wastewater and flue gas (Figure 8). Among trials with RO reject water, DI water had the greatest water flux, followed by cultures grown with simulated wastewater and then the control cultures. A *t*-test was conducted which determined that there was no statistically significant difference (*p* = 0.65) between the water fluxes of the cultures grown with either flue gas or CO_2_-supplemented air, and simulated wastewater (3.0 ± 0.1 and 3.0 ± 0.3 LMH, respectively). The water flux values were relatively low; previous studies achieved 6.71 LMH for comparable conditions.^50^ In 5-h trials, biomass concentrations increased by 40% ± 16% and 40% ± 3% for flue gas and CO_2_-supplemented air conditions, respectively (Figure S4). Previous studies have achieved biomass increases of 4 times the original concentration in 4.5 to 6.5 h.^14^ The control biomass concentration increased by 25% in a single trial.

A preliminary experiment with DI water and 3 M NaCl, as the feed and draw, respectively, achieved the greatest water flux, 6.5 LMH (Figure 8), which was low relative to previous studies that achieved 7 LMH for comparable conditions with 1.2 M NaCl.^50^ Two trials with the control culture opposite 3 M NaCl achieved 3.4 and 3.9 LMH (0.4 h and 2.4 h trials, respectively). Control culture opposite simulated RO reject had water fluxes of 1.9 and 2.5 LMH (5 h and 2.6 h trials, respectively). Trials conducted for 45 min with DI water and simulated RO reject, before and after three 5-h trials indicated that 15 h of operation decreased the water flux capacity of the FO membrane (Figure 8), though no rate decrease was observed across the 5-h spans of each batch trial.

**Figure 9:**
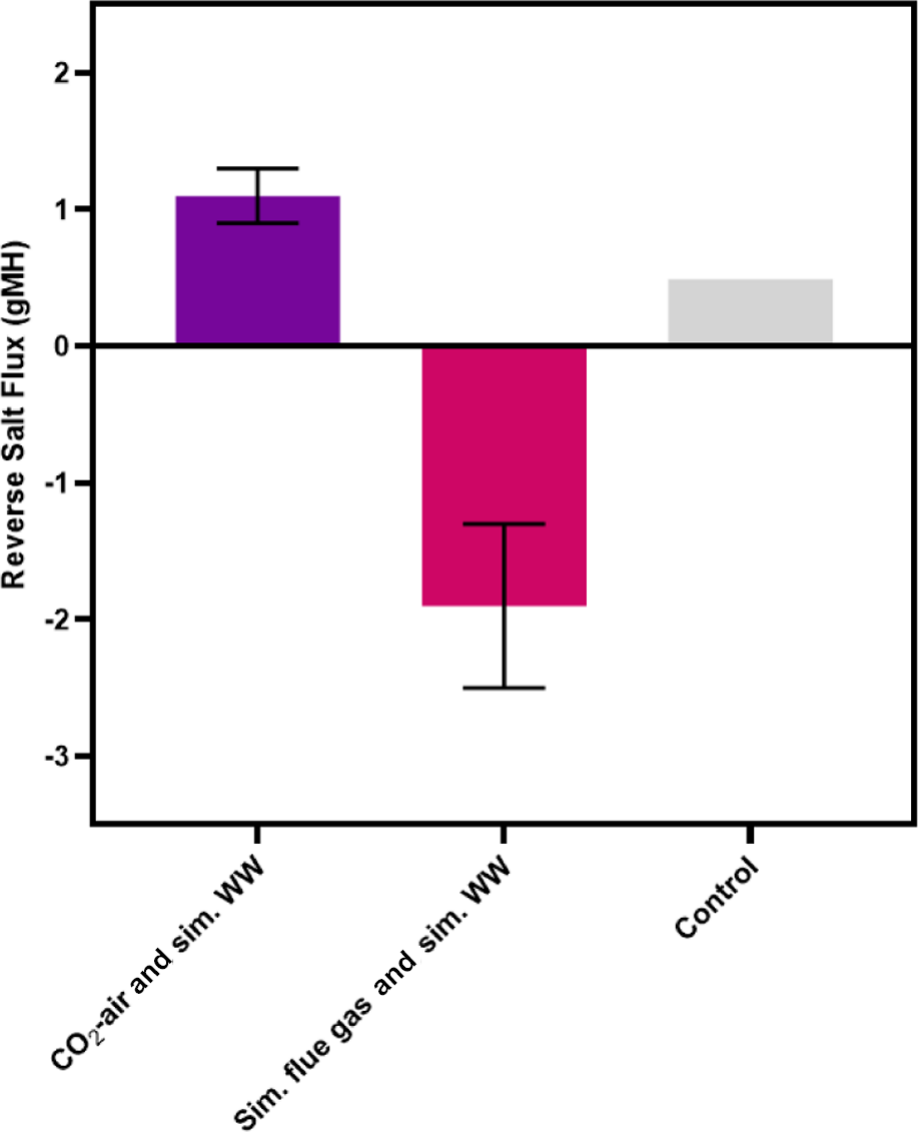
Comparison of the reverse salt flux after 5-h FO trials for three microalgal cultivation conditions: CO_2_-supplemented air and simulated wastewater, simulated flue gas and simulated wastewater, and control. In each case, simulated RO reject water was the draw solution. Error bars represent ±1 standard deviation.

The reverse salt flux was low for all FO trials with each culture condition. The reverse salt fluxes of the cultures grown with simulated flue gas and simulated wastewater, CO_2_-supplemented air and simulated wastewater, and the control were −1.9 ± 0.6, 1.1 ± 0.2, and 0.5 gMH, respectively (Figure 9). Given the osmotic gradient between the draw and feed solutions, it is highly unusual that the reverse salt flux was a negative value for the simulated flue gas condition. During the simulated flue gas cultivated batches, the salt content of the solution would have increased by approximately 1 g L^–1^ due to SO_4_^2–^ accumulation and 0.4 g L^–1^ from base addition (10 mL 1 M NaOH). These increases more than tripled the initial conductivity of the feed solution relative to the CO_2_-supplemented air condition (3.50 ± 0.04 and 1.10 ± 0.35 mS cm^–1^, respectively) and decreased the salinity gradient between the feed and the draw.

### FO Membrane Fouling

**Figure 10:**
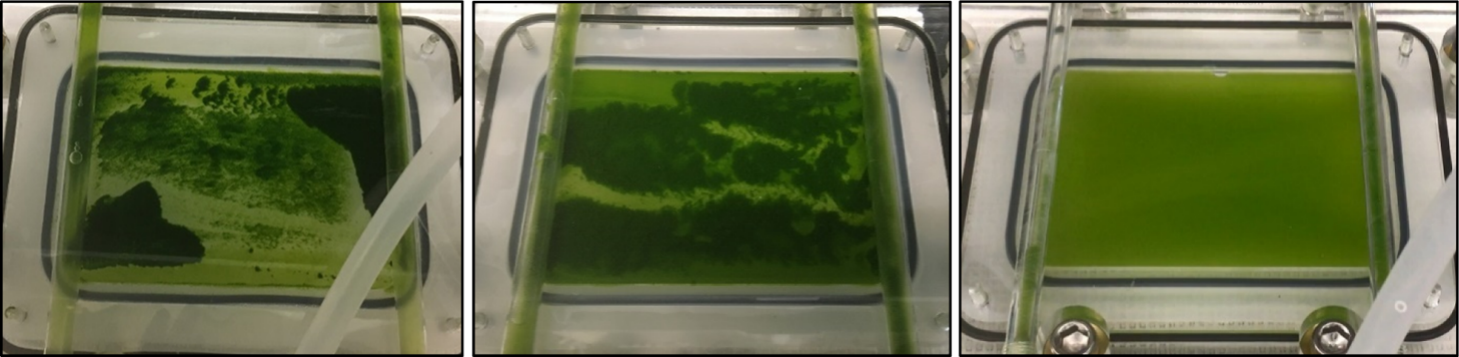
Accumulation of microalgae on the FO membrane during a trial with microalgal culture as the feed (left: simulated flue gas and simulated fertilizer plant wastewater, center: CO_2_-supplemented air and simulated fertilizer plant wastewater, and right: control) and simulated RO reject water as the draw. The microalgal biomass cultivated with simulated flue gas appeared to form much denser flocs than the biomass grown with CO_2_-supplemented air. Control cultures did not flocculate.

Though 5-h FO trials achieved water flux rates of approximately 3 LMH, the potential of this low-energy technology in this scenario was somewhat negated by biomass accumulation within the FO cell (Figure 10). Biomass accumulation within the FO cell was greater for cultures grown with simulated wastewater and/or simulated flue gas, relative to control cultures, because higher concentrations of EPS drove flocculation as microalgae reached this area of lower flow. Floc formation differed between cultures grown with CO_2_-supplemented air and simulated emissions; batches grown with simulated emissions had greater EPS production and formed much denser flocs than those of the batches with only simulated wastewater (Figure 10).

During trials with control culture, biomass did not accumulate within the FO cell. However, the accumulation of biomass in the FO cell was immediate and remarkable even at the start of trials that used simulated wastewater with CO_2_-supplemented air or simulated flue gas (see Supporting Information, Figure S5). Data for this phenomenon were not collected during the first two trials with simulated flue gas cultures because mixing was attempted during the FO trial. However, 60% ± 14% of the microalgal biomass grown with CO_2_-supplemented air collected on the FO membrane. The accumulation of biomass grown with simulated flue gas was 85% (single trial).

Clearly, the sparging of simulated flue gas in the bioreactor stimulated microalgae to produce high EPS concentrations and thus the ability to undergo flocculation and sedimentation. To our knowledge, this is the first report of the effect of simulated flue gas (University of Iowa Power Plant gas concentrations) on the EPS concentrations of microalgae and settleability. Separating microalgae from water in an energy efficient manner is key to the process of harvesting microalgal biomass for beneficial uses and a sustainable, circular economy.

## Conclusions

In this work, a green microalgal species was cultivated and harvested with simulated waste resources. *S. obliquus* removed ammonium rapidly and reached simultaneous N- and P-limitation on Day 5 when cultivated with simulated fertilizer plant wastewater. The flue gas cultivation conditions, in combination with microenvironment ammonia toxicity, caused the microalgae to produce significantly greater quantities of EPS, which in turn promoted good flocculation. The flocs of microalgae grown with simulated fertilizer plant wastewater and CO_2_-supplemented air (without flue gas) were less strongly coagulated and settled much less rapidly. The flocculation was advantageous for rapid settling (especially for the simulated flue gas condition) but disadvantageous to the performance of FO separation trials due to the sedimentation of the algae within the FO cell. For both experimental conditions, FO trials achieved modest water flux rates of 3 LMH, but more than 60% of the biomass accumulated within the FO cell. However, reverse salt fluxes were low.

## Materials and Methods

### Photobioreactor System

A 2 L Sartorius Biostat A bioreactor (Sartorius Stedim, Göttingen, Germany) fitted with two red and blue LED panels (280 μmol m^–2^ s^–1^; Roleadro, San Francisco, CA, USA) served as a pH-stat system and a photobioreactor for microalgae cultivation. Batch studies were conducted in 1.5 L of simulated fertilizer plant wastewater (see Supporting Information, Table S1) at 27 °C, with 10 mM *N*-(2-hydroxyethyl)piperazine-*N*′-ethanesulfonic acid (HEPES) buffer, and pH 6.8 under continuous illumination and at a stir rate of 200 rpm. Constant feedback from the pH-meter controlled the addition of base (1 N NaOH) to maintain the pH setpoint.

The continuously metered gas flows (Masterflex variable-area flowmeter, Cole-Parmer, Vernon Hills, IL, USA) of two custom Praxair cylinders were combined, just before entering the photobioreactor, for a final inlet gas composition of 12% CO_2_, 6% O_2_, 500 ppm SO_2_, 500 ppm CO, 200 ppm NO_2_, and balance N_2_. The toxic nature of the gases (NO_2_, SO_2_, and CO) necessitated extra safety measures.^51^ The reactor was sparged continuously at a total rate of 0.1 L min^–1^ (0.07 vvm).^52^ The concentration of CO_2_ sparged into the reactor was confirmed using GasLab software and a CozIR Wide-Range 0–20% CO_2_ sensor (CM-0123, Gas Sensing Solutions Ltd., Glasgow, UK).

For each batch experiment, the bioreactor was inoculated to optical density at 750 nm (OD_750_) of 0.015 ± 0.005 and sampled daily as each batch progressed from lag to exponential to stationary phase. Biomass values were calculated from a calibration curve relating OD_750_ measurements to cell dry-weight concentrations [OD_750_ = 0.002073 × (CDW in mg L^–1^) + 0.07212].

Microalgal biomass concentrations over time from triplicate batches were fit with a logistic regression and the maximum biomass productivity was calculated from the derivative of the logistic regression at the sigmoid midpoint.

### Inoculum Preparation

*S. obliquus* (SAG 276-1) was obtained from the Culture Collection of Algae at Göttingen University (Göttingen, Germany). Species identity was confirmed through DNA extraction, Sanger sequencing with primers EukA and EukB,^35^ and BLAST sequence search. The *S. obliquus* inoculum was prepared from pure cultures stored on refrigerated 3N-BBM agar slants. Colonies from the slants were used to inoculate 100 mL of sterile 3N-BBM in 500 mL Erlenmeyer flasks capped with foam plugs. Cultures were grown in the flasks for approximately 6 days (the approximate mid-point of the exponential growth phase after transfer from refrigerated stock), at 25 °C and 16:8 h light cycle, before use in bioreactor experiments.

### Control Conditions

Control experiments used threefold nitrogen content BBM,^53^ and Praxair high-purity CO_2_ and Ultra-Zero air to attain 12% CO_2_. The control cultures had the same temperature, pH, gas flow rate, stirring rate, optical density upon inoculation, and HEPES buffer concentration as the experimental cultures. *t*-Tests were used to compare the control and treatment means.

### Nutrient Quantification in Culture Medium

During the batch trials, sulfate, phosphate, ammonia, and nitrate concentrations were measured in daily samples of the culture medium (0.2 μm filtered) using HACH kits SulfaVer 4, TNT846, TNT832, and TNT836, respectively.

### EPS Quantification

Polysaccharides, the dominant component of EPS,^54^ were used as a surrogate to represent the concentration of EPS accumulated in the culture medium when stationary phase was achieved for each culture condition. Well-mixed samples were collected in 50-mL aliquots and then centrifuged for 10 min at 5,000 rpm. The supernatant was collected for triplicate analysis via the anthrone–sulfuric acid method.^55^ Measurements were made at *A*_625nm_, and EPS concentrations were calculated from a calibration curve relating the d-glucose concentration to *A*_625nm_ (see Supporting Information, Figure S6).

### Bulk Settling Experiments

Static column settling tests, modeled after sludge volume index (SVI) tests in the wastewater field, were conducted with a graduated cylinder in triplicate. Microalgal cultures were mixed well (200 rpm) prior to pouring the culture into the graduated cylinder to a height of 33.3 cm. The bulk height of the biomass, grown with either simulated flue gas or CO_2_-supplemented air and simulated fertilizer plant wastewater (containing NH_4_^+^), was observed for a period of 30 min. The same procedure was applied to the culture grown with 12% CO_2_ and Ultra-Zero air in 3N-BBM (control).

Bulk settling for the biomass cultivated with simulated wastewater was best modeled with one-phase exponential decay (eq 3)
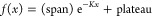
3where *span* is the settling region, *K* is the settling rate, and *plateau* is the minimum compacted bulk height.

Bulk settling for the biomass cultivated with flue gas was best modeled with a two-phase exponential decay (eq 4)
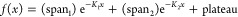
4where *span_1_* is the region dominated by coagulation and rapid settling, *span*_2_ is the region dominated by compaction, *K_1_* is the rapid settling rate, *K_2_* is the compaction rate, and *plateau* is the minimum compacted bulk height.

The fractions of biomass in the settled bulk and the suspended remainder were quantified by measuring the volume and OD_750_ of each portion.

### FO System

A bench-scale acrylic SEPA FO cell (Sterlitech Corporation, Kent, WA, USA) with 140 cm^2^ cellulose triacetate FO membranes (Sterlitech Corporation, Kent, WA, USA), with the active layer facing the feed solution, was used for all FO experiments (see Supporting Information, Figure S7). The feed and draw solutions were circulated through their respective chambers at 80 ± 10 mL min^–1^ during operation. Feed and draw influents and effluents were analyzed before and after operation to determine the water flux and reverse salt flux. Cultures grown with simulated wastewater and either CO_2_-supplemented air or simulated emissions were processed in triplicate 5-h FO experiments for comparison of water fluxes, biomass concentration, and reverse salt fluxes. Between each trial, the membranes were rinsed with DI water.

In our study, simulated RO reject water from seawater desalination served as the draw solution (see Supporting Information, Table S2), opposite the microalgal culture feed solution. The initial salt concentration of the simulated RO reject water was 70.9 g L^–1^.

Preliminary experiments were conducted with combinations of 3 M NaCl or simulated RO reject as draw solutions and DI water or control culture as feed solutions.

### Water and Reverse Salt Flux

The water flux across the FO membrane (from the feed to the draw side) was quantified by tracking the increase in draw solution mass every 10 s during trials with a balance (EJ2000, A&D Engineering Inc., San Jose, CA, USA) attached to a data logger (Simple Data Logger, SmartLUX SARL, Born, Luxembourg). To determine the water flux (in L m^–2^ h^–1^, or LMH), the increase in draw solution mass was divided by the membrane area (0.014 m^2^) and the FO trial duration (in hours).

The reverse salt flux (in g m^–2^ h^–1^, or gMH) across the FO membrane (from the draw to the feed side) was quantified by comparing the conductivity of the feed solutions before and after FO trials via a conductivity probe (Pocket Pro high range conductivity tester, HACH, Loveland, CO, USA). A calibration curve for conductivity and the salt concentration of the simulated RO reject water enabled calculation of salt concentrations from the conductivity measurements (see Supporting Information, Figure S8).

### Concentrating Biomass through FO

The change in microalgal biomass concentrations of the FO feed solutions (batch cultures), pre- and post-FO, was quantified by measuring the OD_750_ of the feed before and after each trial, then calculating the percent increase. When *S. obliquus* was grown with simulated wastewater and either CO_2_-supplemented air or simulated flue gas, the flocculation of the cultures was significant and caused biomass to settle inside the FO cell. The fraction of the biomass that settled within the FO cell was quantified by comparing OD_750_ measurements in the feed bottle before and after a rapid mixing cycle that resuspended the biomass homogeneously.

## Supporting Information Available

Fertilizer plant wastewater formula, RO reject water model composition, EPS calibration curve using the anthrone–sulfuric acid method, conductivity versus simulated RO reject salt concentration calibration curve, FO setup, and control culture growth and nutrient removal graphs (PDF)
