# Supplementary material for: Sustainably Cultivating and Harvesting Microalgae through Sedimentation and Forward Osmosis Using Wastes
Source: ACS Omega. 2021 Jun 25;6(27):17362–71. doi: 10.1021/acsomega.1c01474 (PMC8280685; doi:10.1021/acsomega.1c01474)
Supplement: Supplementary file 1 — ao1c01474_si_001.pdf [file ao1c01474_si_001.pdf]

Supporting Information

Sustainably cultivating and harvesting microalgae through sedimentation and forward osmosis using wastes

Hannah R. Molitor,*† Alyssa K. Schaeffer,† and Jerald L. Schnoor†

† Department of Civil and Environmental Engineering, University of Iowa, 103 S. Capitol St., Iowa City, Iowa 52242, United States

* Email: hannah-molitor@uiowa.edu

(Summary Content: 8 Pages, 8 Figures, 2 Tables)

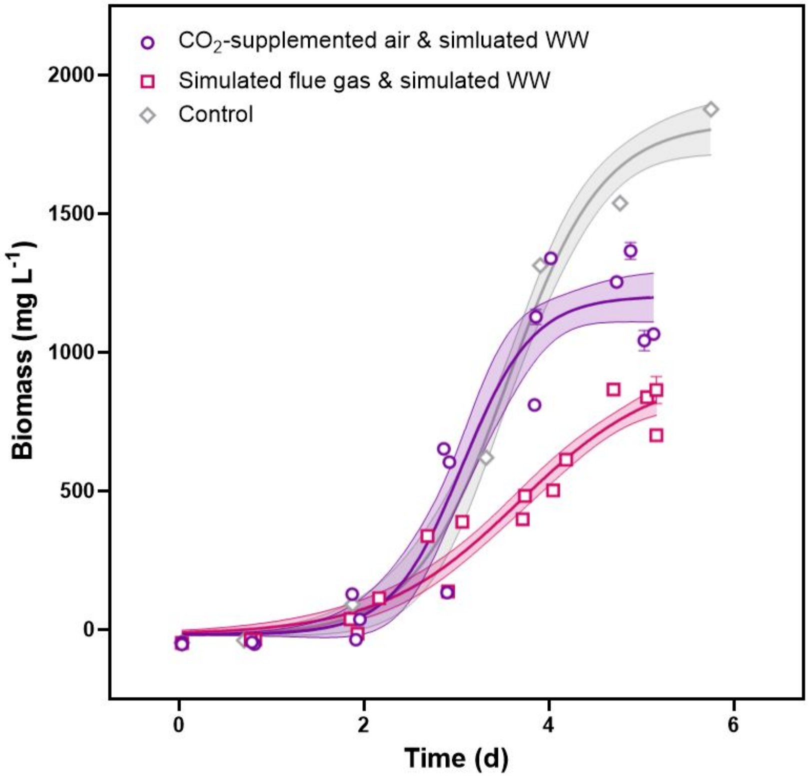

Figure S1.Biomass productivity of *S. obliquus* growth with 1) simulated coal-fired power plant emissions and simulated fertilizer plant wastewater, 2) CO_2- supplemented air and simulated fertilizer plant wastewater, and 3) CO_2- supplemented air and 3N-BBM (control). All points for both triplicate experiments are shown. Vertical error bars represent standard deviation, and the shaded regions represent 95% confidence intervals on the modeled curves.

A control experiment was conducted to ensure that the flocculation observed in the experimental conditions was not an overlying experimental issue.

The control overall biomass productivity was 336 ± 2 mg L^{-1} d^{-1} (single trial). Previous experiments, using 3N-BBM and simulated flue gas, resulted in biomass productivity of 323 ± 2 mg L^{-1} d^{-1}.[32]

The maximum biomass productivity of the control experiment was 900 ± 110 mg L^{-1} d^{-1}. Previous experiments, using 3N-BBM and simulated flue gas, resulted in maximum biomass productivity of 700 ± 40 mg L^{-1} d^{-1}.[32]

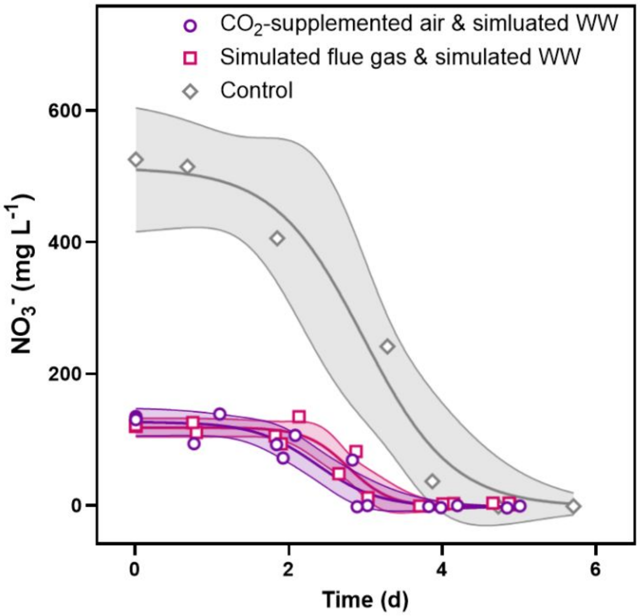

Figure S2.Removal of NO_3^- from culture medium over time for experiments with simulated wastewater and either CO_2- supplemented air or simulated flue gas. Shaded regions represent 95% confidence intervals on the modeled curves. Graph includes control data. In the control, the initial NO_3^- was 547 mg L^{-1} rather than the initial 118 mg L^{-1} NO_3^- of the simulated fertilizer wastewater.

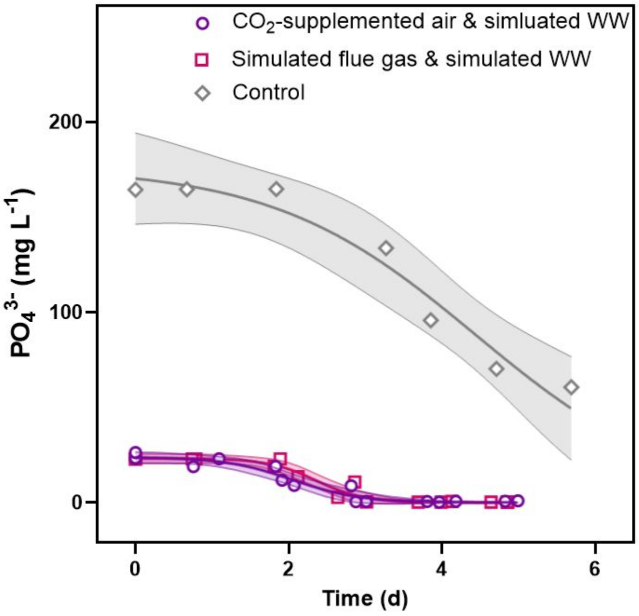

Figure S3. Removal of PO_4^{3-} from culture medium over time for experiments with simulated wastewater and either CO_2- supplemented air or simulated flue gas. Shaded regions represent 95% confidence intervals on the modeled curves. Graph includes control data. In the control, the initial PO_4^{3-} was 164 mg L^{-1} rather than the initial 23 mg L^{-1} PO_4^{3-} of the simulated fertilizer wastewater.

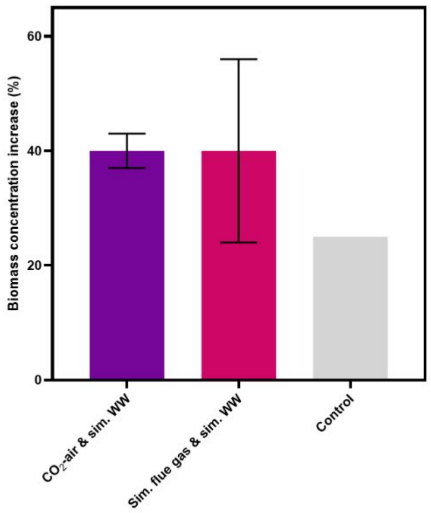

Figure S4. Comparison of the biomass concentration percent increase after 5-hour FO trials for three microalgal cultivation conditions: CO_2-supplemented air and simulated wastewater, simulated flue gas and simulated wastewater, and control. In each case simulated RO reject water was the draw solution. Error bars represent ±1 standard deviation.

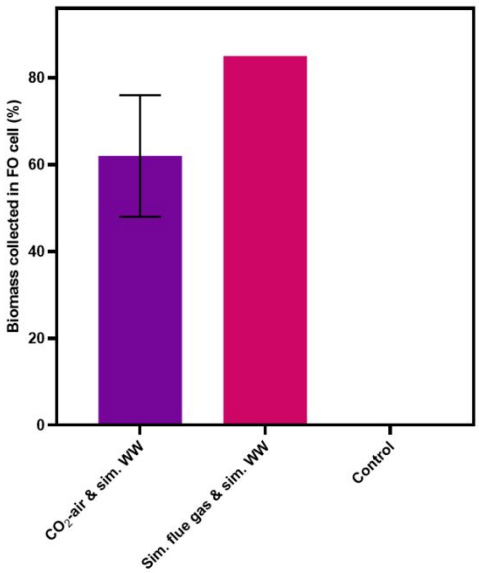

Figure S5. Percent of microalgal biomass collected in the FO cell during trials with simulated RO reject water as the draw solution. Control cultures did not accumulate in the FO cell. Error bars represent ±1 standard deviation.

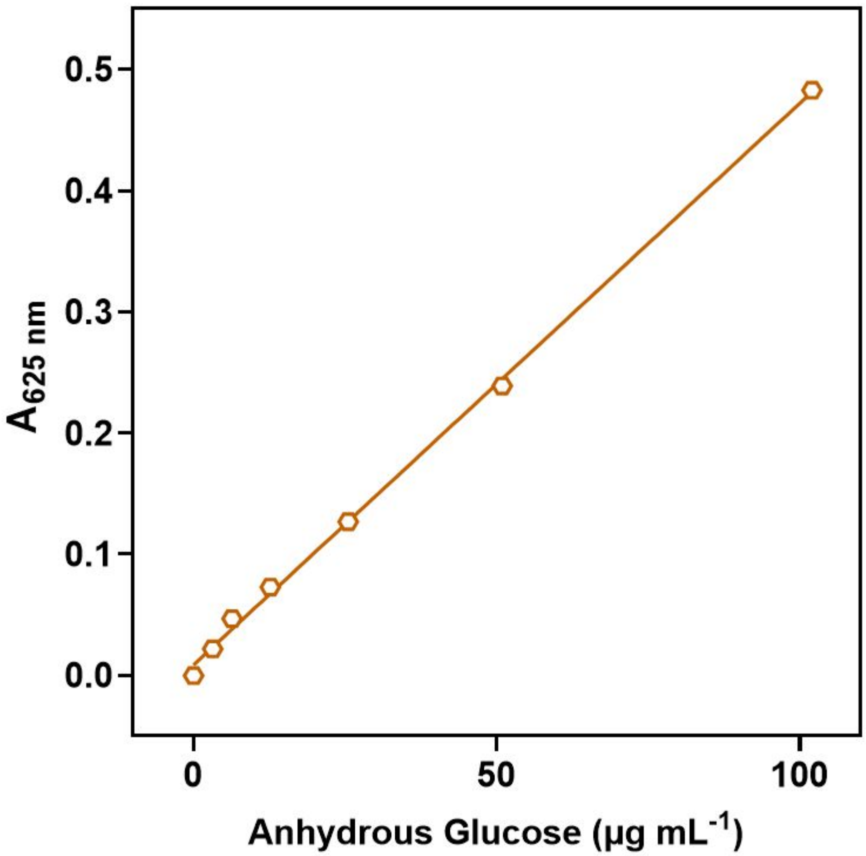

Figure S6. Calibration curve relating anhydrous glucose concentration to absorbance at 625 nm. $y = 0.00463x + 0.0088$. $R^2 = 0.9987$.

Table S1. Simulated fertilizer plant wastewater composition

Compound	Concentration (mol L^{-1})	Concentration (mg L^{-1})
NH_4^+	1.47E-03	26.5
NO_3^-	1.91E-03	118.2
PO_4^{3-}	2.41E-04	22.9
Mg^{2+}	1.52E-04	3.7
Na^+	1.64E-03	37.7
K^+	3.62E-04	14.1
SO_4^{2-}	1.52E-04	14.6
Ca^{2+}	8.50E-05	3.4
Zn^{2+}	1.53E-05	1.0
Mn^{2+}	3.64E-06	0.2
Mo^{6+}	2.47E-06	0.2
Cu^{2+}	3.14E-06	0.2
Co^{2+}	8.42E-07	0.0
B^-	9.23E-05	1.0
Fe^{2+}	8.96E-06	0.5

Simulated fertilizer wastewater composition was based on effluent measurements from a nitrogen fertilizer plant in Vietnam.[7] Phosphate was supplemented to achieve an N:P ratio of 14:1 and half strength Bold's Basal Medium trace nutrients were added.

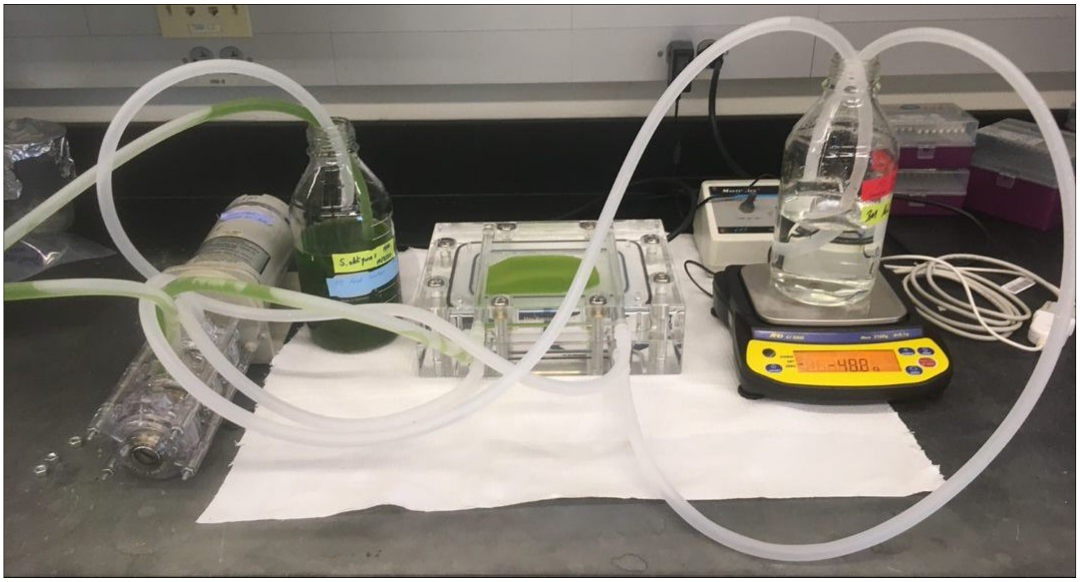

Figure S7. Bench-top forward osmosis system pre-FO trial, as tubing is being primed. The draw solution mass is tracked by a balance that communicates with a data logger.

Table S2. Simulated Reverse Osmosis Reject Water Composition

Salt	Concentration (g L^{-1})
NaCl	46.8
$CaCl_2*H_2O$	2.4
KCl	1.3
$MgCl_2*6H_2O$	8.6
$MgSO_4*7H_2O$	11.8

Simulated RO reject water was based on measurements of major ions in RO reject water from a seawater desalination plant in South Korea.[43]

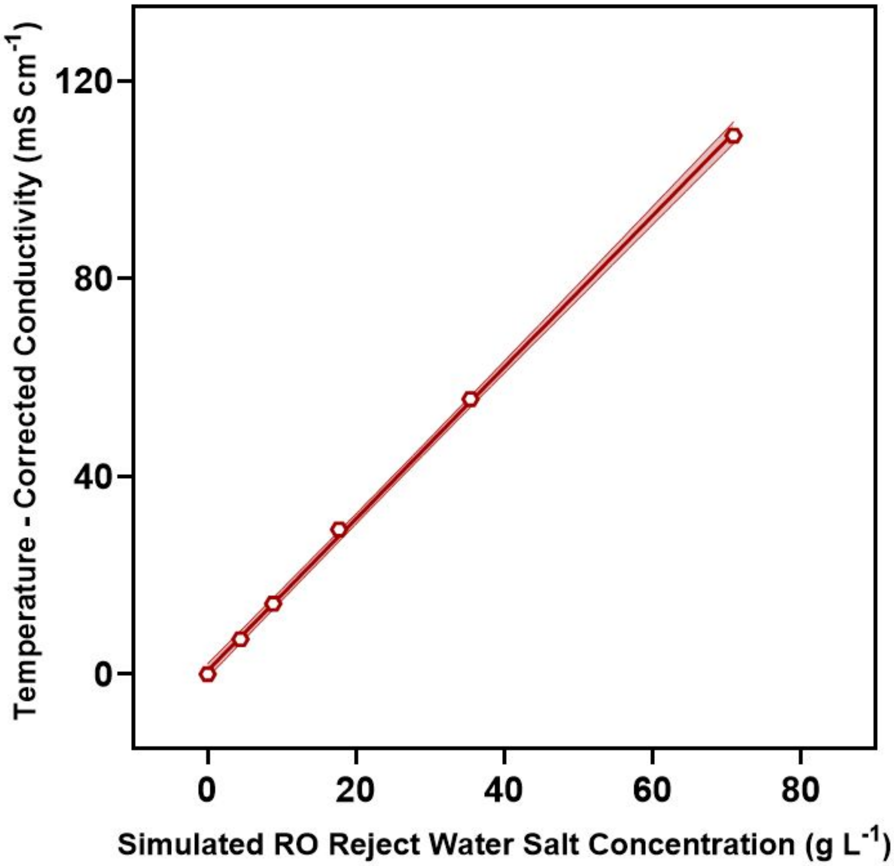

Figure S8. Calibration curve relating the salt concentration of simulated RO reject water to its conductivity at 25 °C. y = 1.534x + 0.7493. R^2 = 0.9996.
